# What Should Be Done To Tackle Ghostwriting in the Medical Literature?

**DOI:** 10.1371/journal.pmed.1000023

**Published:** 2009-02-03

**Authors:** Peter C Gøtzsche, Jerome P Kassirer, Karen L Woolley, Elizabeth Wager, Adam Jacobs, Art Gertel, Cindy Hamilton

## Abstract

Background to the debate: Ghostwriting occurs when someone makes substantial contributions to a manuscript without attribution or disclosure. It is considered bad publication practice in the medical sciences, and some argue it is scientific misconduct. At its extreme, medical ghostwriting involves pharmaceutical companies hiring professional writers to produce papers promoting their products but hiding those contributions and instead naming academic physicians or scientists as the authors. To improve transparency, many editors' associations and journals allow professional medical writers to contribute to the writing of papers without being listed as authors provided their role is acknowledged. This debate examines how best to tackle ghostwriting in the medical literature from the perspectives of a researcher, an editor, and the professional medical writer.

## Abstract 2

In a debate Peter Gøtzsche argues that ghostwriting in medical literature is scientific misconduct, Jerome Kassirer says more evidence is needed, and Karen Woolley and colleagues propose an explicit role for professional medical writers.

## Viewpoint by Peter C. Gøtzsche: Ghostwriting Is Scientific Misconduct and Should Be Handled Accordingly

Scientific communication depends on trust. We should be able to believe what we read, and trust that knowledge when we plan experiments and treat patients. Unfortunately, we cannot. Conflicts of interest are ubiquitous; billions of dollars are being earned undeservedly by drug companies through flaws in research, research articles, reviews, and editorials; and many academic careers have also been built on doubtful evidence. The recent stream of books about the corruptive influence of money on health care research and practice, some of which have been written by editors of our most prestigious journals [1–3], illustrates just how bad the situation is.

Part of the problem is that good names give papers credibility. A colleague once told me that in his country it was more important to know the authors than the methods of a research paper, as some professors lent their names to almost anything if they were well paid. I have seen single-authored meta-analyses on drugs presenting sophisticated analyses that went far beyond the capability of the author, without a word about who did the analyses (and presumably even wrote the paper). Similarly, many drug reviews are unlikely to have been written by the authors, as these professors probably have more important things to do than writing book-length drug reviews in sponsored supplements or peripheral journals that few would ever read and that have no impact factor.

Ghost authorship exists when someone has made substantial contributions to writing a manuscript and this role is not mentioned in the manuscript itself [4]. It often occurs simultaneously with its opposite, guest authorship (sometimes called honorary or gift authorship), where the contributions of the named authors are so small, or nonexistent, that they do not merit authorship [5,6].

Court cases that allowed access to industry files have shown that ghost and guest authorship are common, even in our best journals [5,6]. But misappropriation of authorship is dishonest [4] and is regarded as scientific misconduct in some jurisdictions like Denmark, where the law states that misappropriation of author role, either deliberately or through serious neglect, is a type of scientific misconduct [7]. Regrettably, it is rarely detected. The involved parties have a common interest in secrecy, and junior researchers can ruin their careers if they reveal that the professor did not write the papers that bear his or her name. It is still commonly accepted that department chairs claim authorship of all papers emanating from the department, and newspaper articles celebrating a professor's 60th birthday may note that he or she has written more than 500 papers. The professor may have contributed, but almost certainly wrote only a minority of them.

I have some suggestions that might reduce the prevalence of misappropriated authorship:

1. Most importantly, all journal articles should list the contributions of the authors [8]. This would make it far easier for authors and editors to object before publication, and to document cheating after publication.
2. Editors should explain in their “Instructions to Authors” that ghost and guest writing is scientific misconduct and will be exposed if detected, possibly alerting the authors' academic institutions, and identifying the commercial companies [4]. Editors' associations, such as the Committee on Publication Ethics, the Council of Science Editors, the International Committee of Medical Journal Editors (ICMJE), and the World Association of Medical Editors (WAME), should develop policies that recommend ghostwriting be deemed scientific misconduct.
3. Editors should ask authors to specify who wrote the first draft of the paper (and for research studies, who wrote the protocol and did the statistical analyses [5]), and should contact these people to confirm their contribution if they are not authors. Editors should be particularly careful when manuscripts concern drugs or medical devices.
4. Editors should not accept meaningless statements in the Acknowledgments such as “We thank XX” (without specifying for what) or “XX provided editorial assistance” (a euphemism, usually without affiliation, for “XX from Company YY wrote the paper”).
5. Guidelines on good publication practices for drug companies [9] and medical journals [4,10] should be followed.
6. Authors should retain copies of drafts to facilitate investigations of possible misconduct.
7. To ensure accountability, ethical review committees and drug agencies should not accept protocols that have no named authors, although this is very common [5].
8. To properly document misconduct, journals and PubMed should use the term “misappropriated authorship.” Currently, this type of misconduct is listed under “erratum,” but it is rarely an error. It is usually deliberate.
9. Finally, editors should insist that medical writers be authors. The European Medical Writers Association has stated that writers usually do not qualify for authorship, although their role should be acknowledged [11], and Wager has argued, with reference to the ICMJE criteria, that many writers feel they do not fulfill the principle that authors should be able to take public responsibility for the study [11]. However, these criteria only specify that each author should have participated sufficiently in the work to take public responsibility for appropriate portions of the content [10]. As it is not possible to write a paper without judgment and interpretation of data, which Wager recognizes [11], writers fulfill the authorship criteria [5,10].

To put it simply: if a cook isn't cooking, what is a cook then doing?

## Viewpoint by Jerome P. Kassirer: Ghostwriting Is Difficult To Define, and We Need More Evidence of Its Frequency and Impact

Ghostwriting debases fundamental tenets of the medical profession. It violates authors' personal integrity, responsibility, and accountability. More importantly, ghostwriting threatens the very fabric of science and thus the validity of our medical knowledge, and in doing so it jeopardizes patient care.

Denouncing ghostwriting is easy; defining its variants is not. At the extreme, ghostwriting is easy to define. Perhaps the most egregious example is that of a pharmaceutical company's marketing department promoting one of its products by carefully selecting positive reports and deemphasizing the product's risks, and then paying a well-known academic author to submit the paper for publication without attribution [6]. Few would disagree that this behavior is inappropriate, unprofessional, unacceptable, and potentially dangerous. Lesser degrees of ghost involvement in the writing process are also problematic, such as permitting the commercial sponsor of a clinical trial to collect and hold all of the trial's data, providing exclusive statistical expertise and final tables for publication, drafting a complete manuscript of a study it supported, or insisting contractually on the last say on the final manuscript's content and conclusions.

Other kinds of ghost involvement are more ambiguous. Is it acceptable to hire a science writer to interview a physician and write a paper on that subject, which the physician then calls his or her own? Is it appropriate for a scientist in a company to analyze a portion of evidence, write a draft of the information, and then for another author to incorporate that draft into a manuscript without crediting the scientist? Is it acceptable for a physician-researcher to pay someone to do the same? How much help with writing is okay? Where do we draw the line in some of these fringe areas of ghostwriting?

Because many interactions between academics and industry in developing, testing, and reporting on new products are desirable, such authorship definitions are critical. Unfortunately, there is not a great deal of uniformity among journals (the major publishing gatekeepers) in what constitutes an acceptable contribution. The guidelines of the ICMJE and WAME provide a good start [4,10]. They define an author as someone who has made substantial contributions to conception and design, or acquisition of data, or analysis and interpretation of data; has drafted the article or revised for important intellectual content; and has given approval of the final version. More stringent requirements may be necessary, however. Some journals require that authors have had full control of the primary data, have carried out the statistical tests themselves (e.g., *JAMA*), and have created tables and figures themselves. Others require that each author state the specific contributions they have made to the study or to the writing of the manuscript, and some journals publish these (e.g., *BMJ*, *PLoS Medicine*).

Other journals review components of study protocols that are vulnerable to manipulation by the sponsor and will only publish clinical trials that are registered in a public database endorsed by the ICMJE. Increasingly, journals require that the role of the study sponsor be transparent in manuscripts, and in addition will not accept papers unless the decision to publish is controlled by the researchers. Finally, one former editor, Richard Smith, thinks that the entire peer review system should be scrapped because of excessive industry influence on publishing [12]. He would replace the current system by an open, Web-based disclosure of protocols, data, and statistical assessment, with publication only of systematic reviews based on the study data [13]. These tactics go some way toward stemming ghost involvement in the publication process.

Any additional “cure” for ghostwriting must take into account its frequency and impact. Information from questionnaire studies suggests that authorship in up to 10% of published papers could be attributed to ghostwriters [14–17], although the fraction in industry-sponsored clinical trials in one study was considerably higher [5]. In trying to understand the prevalence, we are stymied because we just don't know what we don't know. Moreover, most of the data in these studies are self-reported, and the exact frequency of company-inspired writing is well hidden. And finally, the impact of ghostwriting is even more difficult to estimate. For this reason, we must be careful not to impose excessive regulations to solve problems that may not be threatening.

Nonetheless, editors of medical journals could devote more effort to define what constitutes appropriate and inappropriate participation in studies and manuscript preparation, especially by companies with products at stake. At the very least, editors can demand transparency. Who were the trial designers? Who were the trial conductors, the researchers, and the data managers? Who did the statistics? Who wrote the manuscript and who signed off on the final draft? Can all authors take public responsibility for their roles?

Overtly biased ghostwritten articles can cause patient harm; others damage the public's trust in both the pharmaceutical industry and the medical profession. Loss of trust may be ghostwriting's major victim. Neither the industry nor the profession can afford further damage to their reputations. Both should “just say no” to ghostwriting.

## Viewpoint by Karen L. Woolley, Elizabeth Wager, Adam Jacobs, Art Gertel, and Cindy Hamilton: Professional Medical Writers Can Be Legitimate Contributors to Manuscripts, But Ghostwriting Is Dishonest and Unacceptable

Professional medical writers have been recognized by medical journal editors as legitimate contributors to manuscripts [4,10], but concerns remain about “ghostwriting,” namely the failure to disclose such contributions. Professional medical writers, who must be distinguished from ghostwriters [18], could be valuable allies to those determined to eradicate ghostwriting. Professional medical writers have communication expertise and health care knowledge, and abide by ethical guidelines for medical writers. They assist authors to prepare documents (e.g., abstracts, slides, posters, and manuscripts), but ensure that the authors control the content and that appropriate disclosures of funding and involvement are made. We believe that professional medical writers can offer unique, and too frequently untapped, insight into how to address ghostwriting. We offer the perspective of professional medical writers on three strategies that have been considered for tackling ghostwriting.

### Strategy #1: Why Don't We Ban Medical Writers?

In 2005, the Editorial Board of the *Clinical Journal of Oncology Nursing* tried to challenge ghostwriting by rejecting “… manuscripts that have been written by medical writers or communication companies” [19]. This strategy has not been embraced by many editors. Instead, the Council of Science Editors, the ICMJE, and WAME strive to discourage ghostwriting by requiring authors to disclose medical writing assistance and funding.

We support disclosure, rather than prohibition. Banning medical writers could have unintended consequences. For example, a ban could intensify the ethical and scientific problem of nonpublication. Only 50% of medical research results may ever be published in full [20], and limited writing time is one of the main reasons for nonpublication [16]. Professional medical writers can help authors avoid nonpublication by completing many of the time-consuming manuscript preparation tasks [18,21]. Encouraging, rather than banning, medical writer involvement may be particularly important for helping authors and sponsors reduce the rate of nonpublication (23%) associated with industry-sponsored clinical trials [22].

Banning medical writers could also reduce the quality of manuscripts, particularly if authors have limited time, manuscript writing experience, English language skills, or awareness of reporting guidelines. Professional medical writers have the specialist skills required to help authors communicate in a clear, concise, and credible manner, and to ensure manuscripts meet journal requirements [9,18,21,23,24]. Banning writers may increase the number of poorly prepared or noncompliant manuscripts—a prospect not likely to be welcomed by editorial staff or peer reviewers.

Just as some researchers need statistical assistance, some researchers need writing assistance. We, like the Council of Science Editors, the ICMJE, and WAME, believe that such assistance should be disclosed, but not banned.

### Strategy #2: Why Don't We Develop More Guidelines?

We don't believe that more guidelines are needed; indeed, we assert that existing guidelines already emphasize the need for appropriate disclosure of writing assistance. The consistency among these guidelines is remarkable, given that they have been developed by different stakeholders, including journal editors [4,10], medical writers [9,18,21,23,24], and industry [25]. Despite these guidelines, the appropriate disclosure of medical writing assistance is low [16]. Many authors don't have the time or inclination to keep up-to-date with guidelines. In some instances, appropriate disclosure may only occur because professional medical writers alert authors to their responsibilities. Banning writers or creating more guidelines could exacerbate an already problematic situation. An alternative strategy is required to tackle ghostwriting.

### Strategy #3: Is There Anything Practical We Can Do?

One of the most practical ways to tackle ghostwriting could be the mandatory use of a checklist that could help editors detect ghostwriting and help authors avoid ghostwriters. We consulted professional medical writers and editors in Europe, North America, and the Asia Pacific region to develop a checklist (Table 1) that could be completed by all authors who used medical writers.

**Table 1:**
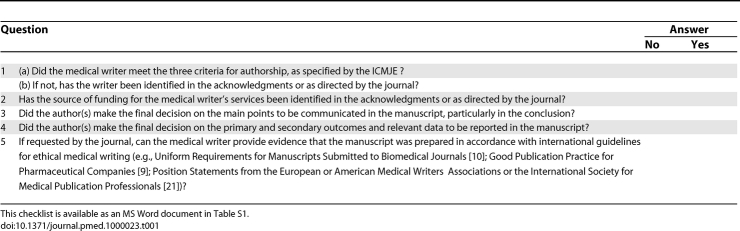
Checklist for Authors Using Medical Writers: A Practical Tool to Discourage Ghostwriting Professional medical writers can be legitimate contributors to manuscripts, but ghostwriting is dishonest and unacceptable. **Authors:** If a medical writer contributed to the preparation of your manuscript, you must answer the questions below.

This checklist prompts authors to acknowledge professional medical writers and their funding source, to confirm that the authors controlled the main points, outcomes, and data reported in the manuscript, and to verify that medical writers could provide evidence that guidelines on ethical writing practices were followed. The checklist balances brevity with utility. Editors could always ask authors additional questions.

The checklist, which could be included in journals' “Instructions to Authors,” could help editors encourage appropriate disclosure of writing assistance, as well as raise awareness of existing guidelines. The checklist is a logical extension of journal editors' gatekeeping role. By putting the onus of use on authors, the checklist could be implemented quickly and without the need for extensive resources. The checklist would also provide sponsors and professional medical writers with a means of documenting appropriate medical writing use. Indeed, an audit trail of appropriate interactions between authors and professional medical writers should be available. Organizations trying to eradicate ghostwriting could educate their members about the checklist.

In conclusion, we believe the debate about ghostwriting needs to shift from whether authors used writers to whether writing assistance was appropriate and adequately disclosed. Professional medical writers are trained to provide appropriate assistance and to insist on disclosure. Since professional medical writers work with experienced and inexperienced authors from around the world on a daily basis, they could be valuable allies in the efforts to tackle ghostwriting.

## Supporting Information

Table S1 Checklist for Authors Using Medical Writers (32 KB DOC).

## Abbreviations

ICMJE: International Committee of Medical Journal Editors
WAME: World Association of Medical Editors
