# Supplementary material for: What Should Be Done To Tackle Ghostwriting in the Medical Literature?
Source: PLoS Med. 2009 Feb 3;6(2):e1000023. doi: 10.1371/journal.pmed.1000023 (PMC2634793; doi:10.1371/journal.pmed.1000023)
Supplement: Supplementary file 1 [file pmed.1000023.st001.doc]

**Table S1. Checklist for Authors Using Medical Writers: A Practical Tool to Discourage Ghostwriting**

Professional medical writers can be legitimate contributors to manuscripts, but ghostwriting is dishonest and unacceptable.

**Authors: If a medical writer contributed to the preparation of your manuscript, you must answer the questions below.**

| **Question** | | **Answer** | |
| --- | --- | --- | --- |
|  |  | **No** | **Yes** |
| 1 | (a) Did the medical writer meet the three criteria for authorship, as specified by the ICMJE ? |  |  |
|  | (b) If not, has the writer been identified in the acknowledgments or as directed by the journal? |  |  |
| 2 | Has the source of funding for the medical writer’s services been identified in the acknowledgments or as directed by the journal? |  |  |
| 3 | Did the author(s) make the final decision on the main points to be communicated in the manuscript, particularly in the conclusion? |  |  |
| 4 | Did the author(s) make the final decision on the primary and secondary outcomes and relevant data to be reported in the manuscript? |  |  |
| 5 | If requested by the journal, can the medical writer provide evidence that the manuscript was prepared in accordance with international guidelines for ethical medical writing (e.g., Uniform Requirements for Manuscripts Submitted to Biomedical Journals [10]; Good Publication Practice for Pharmaceutical Companies [9]; Position Statements from the European or American Medical Writers Associations or the International Society for Medical Publication Professionals [21])? |  |  |
